# Supplementary material for: Transcranial magnetic stimulation induced early silent period and rebound activity re-examined
Source: PLoS One. 2019 Dec 4;14(12):e0225535. doi: 10.1371/journal.pone.0225535 (PMC6892484; doi:10.1371/journal.pone.0225535)
Supplement: Attachment — Submitted filename: Response to Reviewers v2.docx [file pone.0225535.s001.docx]

**Response to Reviewers**

TRANSCRANIAL MAGNETIC STIMULATION INDUCED EARLY SILENT PERIOD AND REBOUND ACTIVITY RE-EXAMINED

Reviewer #2

The following inclusion just glosses over this point “Previously proposed mechanisms for the cortical silent period have focused on theories regarding cortical inhibition [7, 10, 45, 46].” Actually, I think that in the spirit of transparent/clear science, it is absolutely critical to more clearly and explicitly acknowledge that cortical inhibitory mechanisms contribute to the late CSP, in particular, it should be specifically stated that there is clear evidence that epidural volleys are reduced in amplitude during the CSP (Chen et al, 1999) and that Pierantozzi et al., 2004 concluded that the drug effects they observed were not driven by peripheral effects – this indicates that the late cortical component of the CSP is likely an inhibitory phenomenon mediated by GABA. CSP duration is typically in the order of 100-200ms (Chin et al., Brain Res, 2012) and the time-course of spinal contributions has been partially illustrated using brainstem stimulation (Inglhilleri; 1993; JPhysiol). This leaves plenty of scope for the authors to still demonstrate the mechanisms of the spinal contribution and does not detract from this.

A paragraph to make the contribution of the cortical inhibitory mechanisms to the late CSP clearer was included in line with the suggestions of the Reviewer in Page 26.

-Title: “Transcranial magnetic stimulation induced *early* silent period and rebound activity re-examined”.

-Abstract – “Our aim is to better characterize the *early* CSP phenomena by combining various analysis tools on firing motor units.”

“Discharge rate analysis, however, revealed not three, but just two events with distinct time courses; a long-lasting excitatory period (71.2 ± 9.0 ms for TA and 42.1 ± 11.2 ms for APB) and a long-latency inhibitory period.” - *insert total duration at end of sentence (i.e. around 46ms). The early phase of the CSP is conventionally considered as the first 50ms, whereas the remainder up to several hundred ms is considered mostly cortical. I think the article is still not making this distinction sufficiently clear.

-In the discussion, the following header could be retitled as follows for clarity: “*Early* cortical silent period may represent a net excitatory postsynaptic potential”

“This limitation also restricted us from directly comparing the MEP and CSP sizes and durations with the literature as they have used much stronger stimulus intensities than this study. *This means our analysis was constrained to the first 50ms of the CSP, whereas CSP duration is typically in the order of 100-200ms.*”

All included.
